# Acute Severe Cholecystitis in a Patient With Acute Myocardial Infarction and Heart Failure: A Case Report

**DOI:** 10.7759/cureus.94717

**Published:** 2025-10-16

**Authors:** Mahmoud Abdulkareem, Rayan Y Aljaser, Ahmed N AlKhaytan, Abdulaziz M Algethami, Abdulmalik Y Aldebasi, Fahid A Almotairi

**Affiliations:** 1 General Surgery, King Fahad Specialist Hospital, Buraydah, SAU; 2 College of Medicine, Qassim University, Buraydah, SAU

**Keywords:** acute myocardial infarction, acute severe cholecystitis, gallbladder perforation, heart failure, multidisciplinary approach, open cholecystectomy, percutaneous coronary intervention, three-vessel coronary artery disease

## Abstract

Acute severe cholecystitis (ASC) is an advanced inflammatory and infectious disease of the gallbladder, often requiring immediate surgical intervention. In this case, we will discuss the management of a 57-year-old male with a history of recent inferior wall myocardial infarction (MI), heart failure (HF) with a left ventricular ejection fraction (LVEF) of 40-45%, and severe three-vessel coronary artery disease (3VCAD). The patient case presented to the surgical team at the King Fahad Specialist Hospital (KFSH) with acute cholecystitis complicated by gallbladder perforation and a pericholecystic abscess, confirmed through computed tomography (CT). With the patient's cardiac status, it was a difficult decision to operate immediately due to the potential for hemodynamic instability under general anesthesia. Initially, we considered a conservative approach with intravenous antibiotics, followed by ultrasound-guided percutaneous drainage of the abscess. Unfortunately, due to the ineffectiveness of the drainage and the increasing risk of sepsis, a multidisciplinary consultation was held, which arrived at a consensus to perform an open cholecystectomy. The surgical procedure was successful, with no complications. After 10 days, we conducted a percutaneous coronary intervention (PCI), involving stenting of the right coronary artery (RCA) and left circumflex artery (LCX), and balloon angioplasty of the left anterior descending artery (LAD), to improve the cardiac blood flow. The patient was discharged in a stable condition 12 days after the cholecystectomy. In this paper, we aim to highlight the complex management approach to such high-risk scenarios and achieving a positive patient outcome.

## Introduction

Acute severe cholecystitis (ASC) is an advanced inflammatory and infectious disease of the gallbladder, often requiring immediate surgical intervention [1]. The risk of gallbladder perforation substantially increases the chances of sepsis and peritonitis, thus requiring swift and suitable intervention [2]. On the other hand, acute coronary comorbidities like recent acute myocardial infarction (MI) or heart failure (HF) add significant risks to the surgery, such as hemodynamic instability and cardiac arrest under anesthesia [3]. It has been reported that there is an increased risk of major adverse cardiac events (MACEs) following non-cardiac surgeries done within six weeks after receiving a coronary stent [4]. In this case report, we describe the treatment undertaken for a 57-year-old male with ASC, gallbladder perforation with large pericholecystic abscess, along with a recent inferior wall MI, HF, and severe three-vessel coronary artery disease (3VCAD). The merging of these pivotal conditions is uncommon, which creates distinct diagnostic and treatment difficulties [5]. This case illustrates the challenges of managing a severe infection with severe cardiac comorbidities and emphasizes the need for a multidisciplinary approach to improve patient outcomes in critically complex situations [6].

## Case presentation

A 57-year-old male, an active smoker. with a past medical history of diabetes, hypertension, peripheral vascular disease that led to amputation of his left 4th and 5th toes, and a cerebrovascular accident (CVA) resulting in right parietal and right temporo-occipital lobe infarction, from which he had made a full neurological recovery two years and six months ago. We admitted the patient on September 15, 2024, with an acute inferior wall myocardial infarction (MI). After conducting a coronary angiography (CAG), we discovered a severe three-vessel coronary artery disease (3VCAD), a left main artery (LM) with minor atheroma, a left anterior descending artery (LAD) with mid 80-90% stenosis and an ostial 80% stenosis in the first diagonal branch (D1), a left circumflex artery (LCX) with subtotal occlusion at the mid-segment and total distal occlusion, and a right coronary artery (RCA) with total occlusion at the mid-segment.

Regarding his cardiac status, the patient has heart failure (HF) with a left ventricular ejection fraction (LVEF) of 40-45%, accompanied by moderate ischemic mitral regurgitation confirmed by transoesophageal echocardiography (TEE). There weren’t any significant abdominal symptoms at the time of his MI presentation. Our initial management plan was focused on stabilizing the patient's MI and then initiating a coronary artery bypass grafting (CABG) procedure to address the severe coronary artery disease. 

Approximately eleven days after his MI, the patient developed severe right upper quadrant pain, accompanied by fever and nausea. Physical examination revealed significant tenderness in the epigastric and right upper quadrant regions, with guarding. 

Laboratory investigations revealed elevated laboratory values (Table 1). 

**Table 1:** Elevated laboratory values *Reference ranges may vary slightly between laboratories. Abbreviations: ↑ = Elevated; WBC = White blood cell count; AST = Aspartate aminotransferase; ALT = Alanine aminotransferase; ALP = Alkaline phosphatase.

Test	Result	Reference Range*	Interpretation
WBC	28,000/µL	4,000–11,000/µL	↑ Elevated
AST	59 U/L	10–40 U/L	↑ Elevated
ALT	114 U/L	7–56 U/L	↑ Elevated
ALP	146 U/L	44–120 U/L	↑ Elevated
Total bilirubin	7.71 mg/dL	0.3–1.2 mg/dL	↑ Elevated

**Figure 1:**
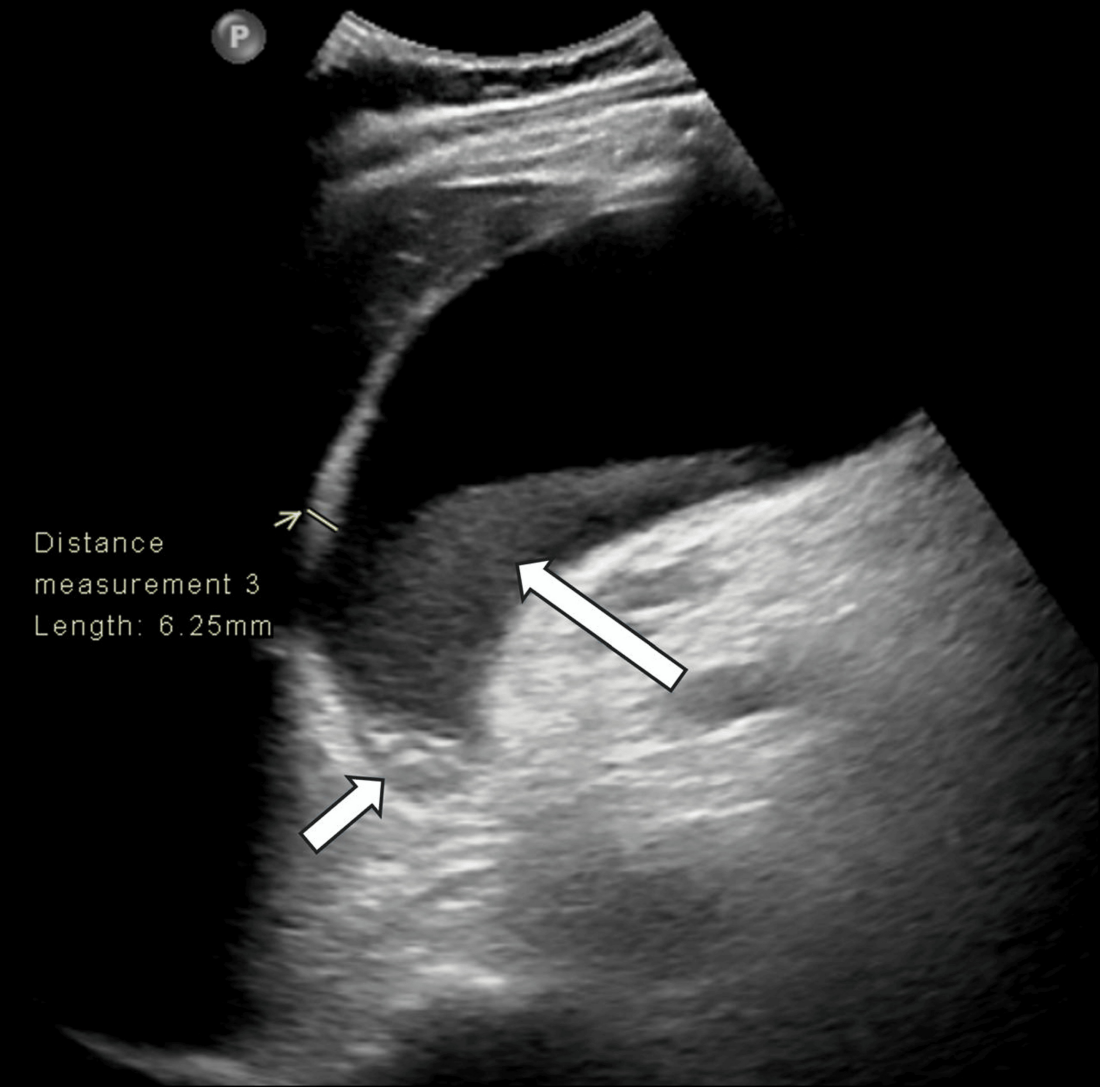
Abdominal ultrasound Abdominal ultrasound showing an over-distended gallbladder (11 cm in the long axis) with irregular wall thickening (up to 6 mm), sludge (long white arrow), calculi (short white arrow) impacted at the gallbladder neck, and positive sonographic Murphy sign. There was no associated biliary dilatation or stones. The appearance is consistent with noncomplicated acute calculous cholecystitis.

**Figure 2:**
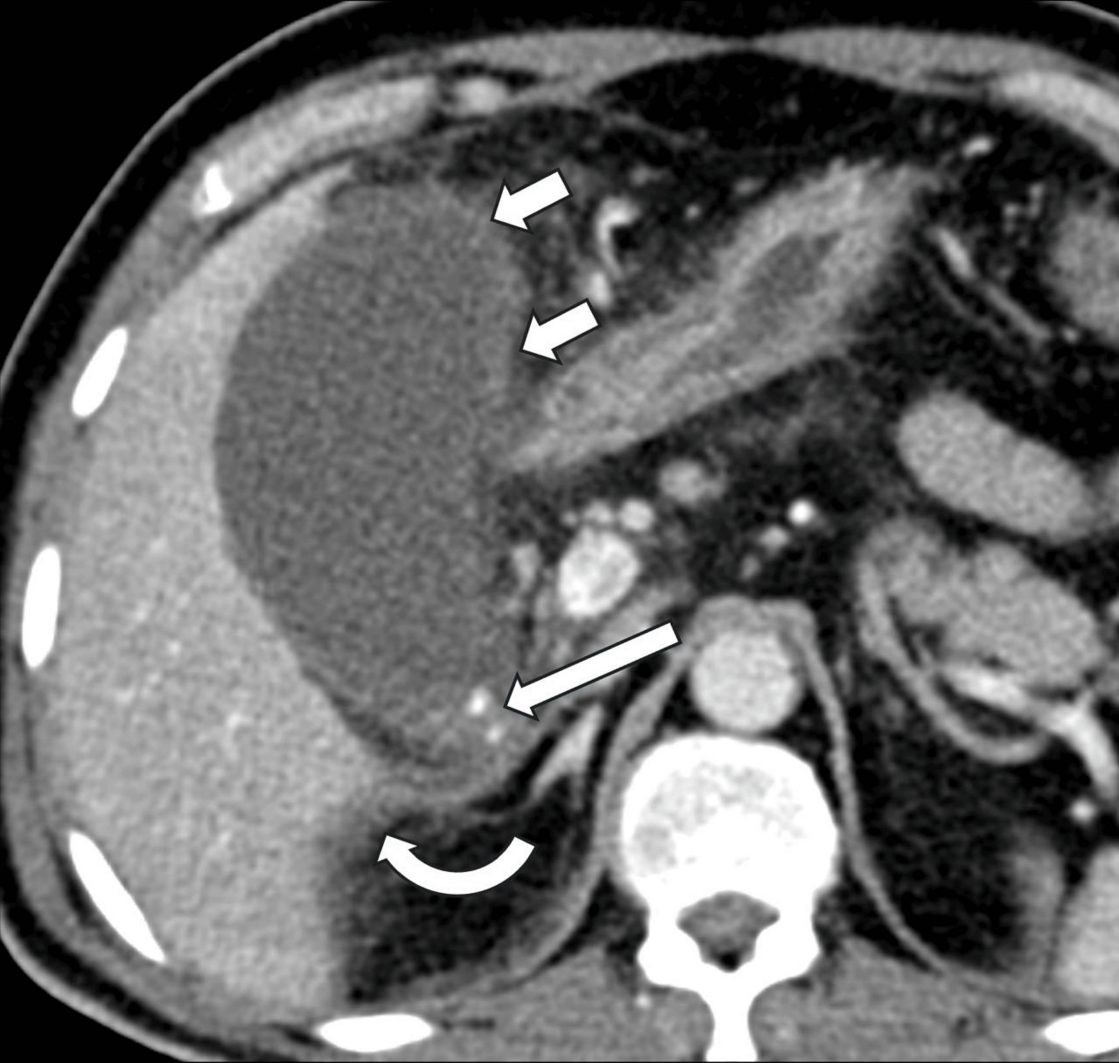
Contrast-enhanced abdominal CT scan (axial image) Contrast-enhanced abdominal CT scan (axial image) one day after ultrasound showing an over-distended gallbladder with sludge and stones (long arrow) at the gallbladder neck. The gallbladder wall is non-enhancing and irregular (short arrows), with probable micro-perforation. There was no mural or luminal gas. Adjacent fat stranding and traces of free fluid are also noted (curved arrow).  The appearance is consistent with gangrenous cholecystitis.

An initial abdominal ultrasound suggested noncomplicated acute calculous cholecystitis (Figure 1). The following day, we diagnosed the patient with acute severe cholecystitis (ASC) after performing a computed tomography (CT) scan of the abdomen, which revealed findings consistent with gangrenous cholecystitis (Figure 2). The patient did not report any concurrent cardiac symptoms, such as chest pain or worsening shortness of breath, at that time. 

Management and outcome

With the patient's condition and risk factors, immediate surgical intervention for the acute cholecystitis was contraindicated due to the elevated risk of hemodynamic collapse under general anesthesia. Initially, we adopted a conservative management plan, which goes as follows: nil per os (NPO) status, intravenous fluids (IVF), analgesics for pain control, and broad-spectrum intravenous antibiotics (piperacillin-tazobactam 4.5 g every 8 hours) to address the infection. The patient’s clinical condition showed signs of deterioration, with persistent fever, worsening leukocytosis (WBC count initially 28,200/µL, later fluctuating to 23,400/µL), and an increasing size of the peri-cholecystic abscess observed on follow-up imaging. 

**Figure 3:**
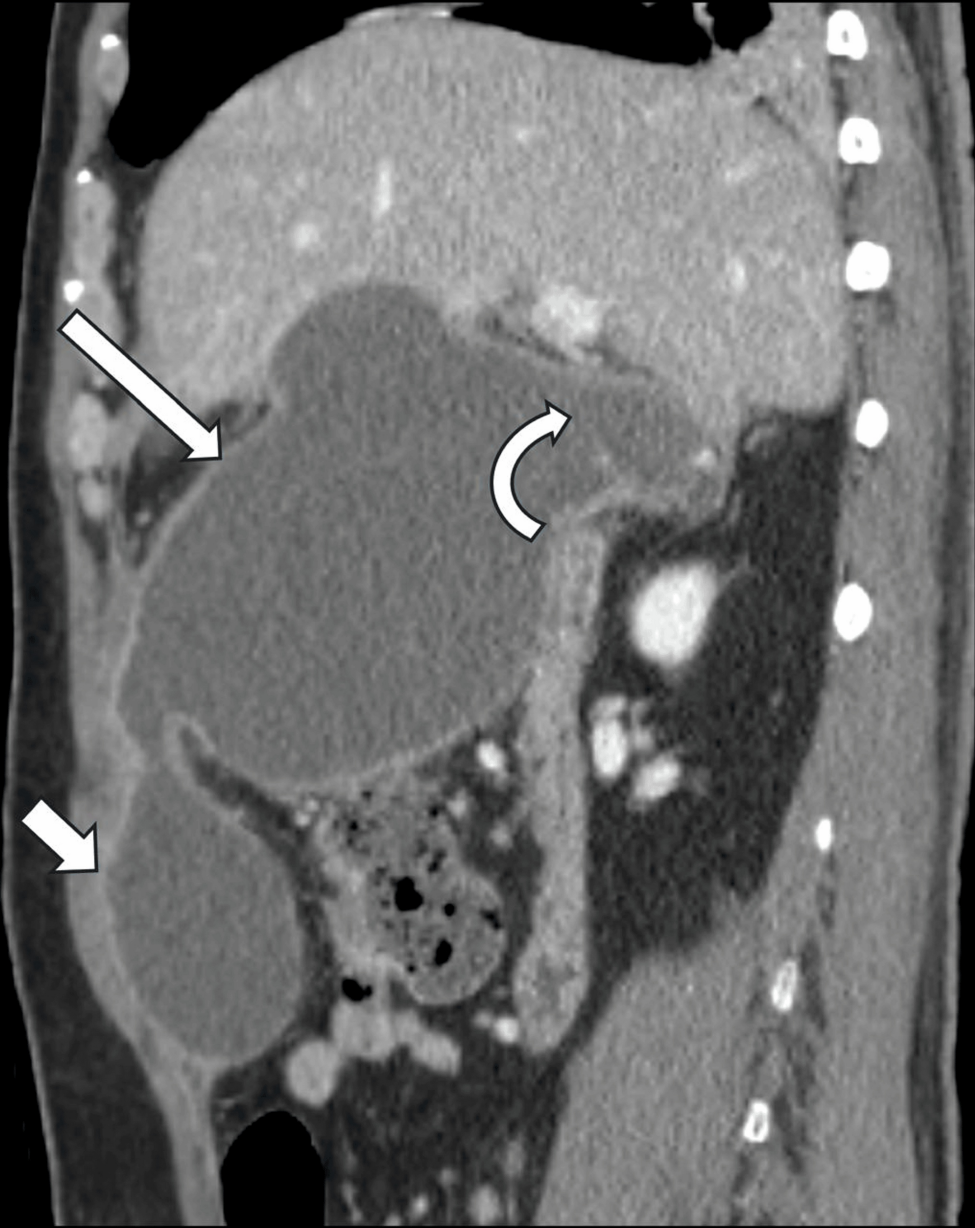
Follow-up contrast-enhanced abdominal CT scan (sagittal image) Follow-up contrast-enhanced abdominal CT scan (sagittal image) showing gallbladder wall defect (curved arrow) associated with two large interconnected rim-enhancing extrahepatic fluid collections (long and short arrows). The larger fluid collection measures 9.8 x 13.5 x 15.7 cm. The appearance is consistent with perforated cholecystitis with extrahepatic infected bilomas/abscesses.

Three weeks after the onset of cholecystitis, a repeated CT scan of the abdomen revealed the development of two large, communicated locules of fluid collections in the peri-cholecystic region, with the largest measuring (9.8 x 13.5 x 15.7 cm), exerting a significant mass effect on the surrounding abdominal structures (Figure 3). The interventional radiology (IR) team had to perform an ultrasound-guided percutaneous drainage of the abscess on the same day. An 8.5 Fr pigtail catheter was inserted to drain the largest fluid collection. After the initial drainage of purulent fluid, the catheter became progressively ineffective, draining less than 20 mL in a 24-hour period by the 15th day of catheter insertion, which may be caused by loculation or blockage within the abscess cavity.

An extensive multidisciplinary discussion had to be held, involving the general surgery team, cardiology, cardiothoracic surgery, cardiac anesthesiology, and ICU consultants. The decision was made to proceed with surgical intervention as a life-saving measure to prevent sepsis, peritonitis, and abscess progression. The surgical decision had to be made considering the ineffective percutaneous drainage over the past two weeks and the increasing risk of life-threatening complications. The necessity of addressing the intra-abdominal issue was prioritized over the previously planned coronary artery bypass grafting (CABG) due to its urgency. The significant risks associated with the patient’s cardiac status were carefully considered while the surgical considerations were taking place, and all necessary precautions to mitigate perioperative risks were planned. The potential mortality associated with both continued medical management and surgical intervention was explained to the patient and his family, and high-risk consent was obtained. After 5 weeks of the initial onset of cholecystitis, the surgery was performed according to the recommendations of the multidisciplinary team, which considered the surgical intervention as a life-saving procedure that needed to be performed as early as possible. 

**Figure 4:**
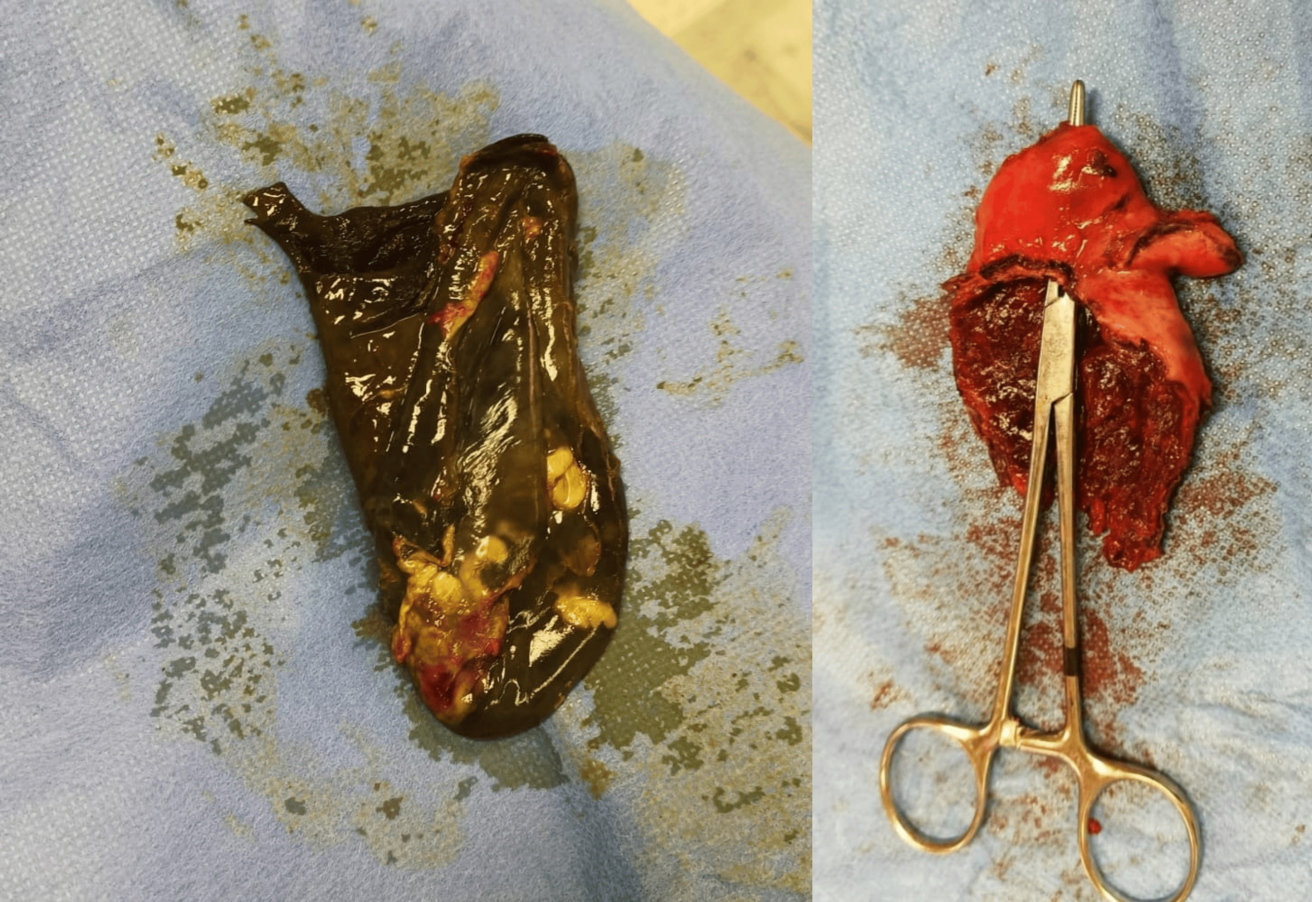
Intraoperative images Intraoperative photograph of the resected gallbladder showing perforation and the presence of gallstones and purulent material. Histopathological report of the resected gallbladder revealed acute necrotizing cholecystitis with extensive hemorrhage and neutrophilic infiltration.

We preferred an open cholecystectomy (laparotomy) approach to minimize potential cardiac stress associated with laparoscopic insufflation, which could exacerbate hemodynamic instability, considering the patient’s compromised cardiac function. The surgical procedure was performed by experienced senior surgeons with the goal of minimizing operative time, as strongly recommended by the cardiac anesthesia team. Upon abdominal exploration, the gallbladder was perforated with a large, walled-off abscess cavity, which was drained. The perforated and inflamed gallbladder was then removed completely. Histopathological report of the resected gallbladder revealed acute necrotizing cholecystitis with extensive hemorrhage and neutrophilic infiltration (Figure 4).

The surgical procedure was successful and completed with the patient in a stable status without requiring any intraoperative blood transfusions. Postoperatively, he was closely monitored in the coronary care unit (CCU). Four days after the surgery, his diet was gradually advanced to a soft, low-salt, fat-free regimen, and his indwelling Foley catheter was removed without complications. Intravenous antibiotics (piperacillin-tazobactam) were continued postoperatively for a total duration of 7 weeks.

Follow-up after the procedure

As planned, the patient underwent percutaneous coronary intervention (PCI) on the 10th day after the surgery. The procedure involved the following: (1) Percutaneous coronary intervention (PCI) to the chronic total occlusion (CTO) of the right coronary artery (RCA) with a 2.75 x 48 mm XIENCE™ stent (XIENCE™ Everolimus Eluting Coronary Stent System; Abbott Vascular, Santa Clara, USA), guided by intravascular ultrasound (IVUS), resulting in an excellent outcome; (2) Percutaneous transluminal coronary angioplasty (PTCA) to the LAD with a 2.5 x 20 mm balloon; and (3) Percutaneous coronary intervention (PCI) to the LCX with a 2.75 x 48 mm Xience stent, guided and optimized by IVUS, also with an excellent result.

The PCI procedure was accomplished through femoral artery access without any complications, signs of bleeding or hematoma at the access, Post-PCI laboratory results showed improvement in inflammatory markers (WBC 13,000/µL), stable haemoglobin (10 g/dL), adequate platelet count (313,000/µL), stable renal function (creatinine 78 µmol/L), and normalized liver enzymes (aspartate aminotransferase (AST) 29 U/L) (Table 2). 

**Table 2:** Laboratory Investigations Before and After PCI. *Reference ranges may vary slightly between laboratories. Abbreviations: ↑ = Elevated; WBC = White blood cell count; AST = Aspartate aminotransferase; ALT = Alanine aminotransferase; ALP = Alkaline phosphatase; PCI = Percutaneous Coronary Intervention.

Test	Initial Result	Post-PCI Result	Reference Range*	Interpretation
WBC (µL)	28,000	13,000	4,000–11,000	Elevated → Improved
Hemoglobin (g/dL)	—	10	13–17 (male)	Stable (low-normal)
Platelets (µL)	—	313,000	150,000–450,000	Adequate
Creatinine (µmol/L)	—	78	60–110	Stable renal function
AST (U/L)	59	29	10–40	Elevated → Normalized
ALT (U/L)	114	—	7–56	Elevated
ALP (U/L)	146	—	44–120	Elevated
Total Bilirubin (mg/dL)	7.71	—	0.3–1.2	Elevated

The patient’s overall clinical condition improved significantly. He was ambulating with assistance and reported a resolution of his abdominal pain. He was discharged in a stable condition on the 12th day after the surgery, with a medical prescription including aspirin, clopidogrel, a beta-blocker, an angiotensin-converting enzyme (ACE) inhibitor, insulin, and analgesics as needed. Furthermore, we provided him with detailed instructions regarding his wound care, medication adherence, and the importance of outpatient follow-up appointments with the cardiology and general surgery teams.

## Discussion

Our case emphasizes the complex management plan of acute severe cholecystitis (ASC) complicated by gallbladder perforation in a patient with a recent acute myocardial infarction (MI), underlying heart failure (HF), and severe three-vessel coronary artery disease (3VCAD). 

Gallbladder perforation, a serious complication occurring in 2-11% of ASC cases, is associated with a significant rise in both morbidity and mortality because of the elevated risk of sepsis and peritonitis [2]. The presence of serious cardiac comorbidities in our patient, including a reduced left ventricular ejection fraction (LVEF) of 40-45% along with moderate ischemic mitral regurgitation, increased the risk of hemodynamic collapse under general anesthesia, thereby making surgical intervention exceptionally challenging [3]. 

Our initial aim was to stabilize the patient and reduce the need for immediate surgical intervention through adopting a conservative management approach that adheres to the Tokyo Guidelines (TG18) for the management of high-risk patients with acute cholecystitis, which recommended the use of a broad-spectrum antibiotic (piperacillin-tazobactam in our case) and keeping the patient on NPO status [7]. 

A more aggressive interventional strategy became necessary when a follow-up CT scan showed progression of the infection to a large, multiloculated pericholecystic abscess (9.8 x 13.5 x 15.7 cm). This led us to advance with the suggested method for handling infected fluid collections in high-risk surgical candidates, with a percutaneous drainage under ultrasound guidance that was ineffective, yielding minimal drainage (<20 mL per day) despite proper catheter placement. This limited drainage was most likely due to the multiloculated nature of the abscess cavity and the presence of viscous biliary sludge and gallstones within the collection [8]. 

Following a comprehensive multidisciplinary consultation, with all involved specialties, recognizing the life-threatening nature of the uncontrolled infection and the high risks of sepsis, peritonitis, and further abscess progression, we had no choice but to proceed with an open cholecystectomy procedure, especially with the dedicated support of our decision from anesthesia team, which reviewed the case and further highlighted the increasing risks associated with the ineffective percutaneous drainage and the potential for a rapid deterioration of the patient’s health [9]. 

Choosing an open surgical approach instead of a laparoscopic technique was a critical consideration; this was due to concerns of increased intra-abdominal pressure during laparoscopic insufflation to negatively affect cardiac preload and potentially exacerbate heart failure, particularly in a patient with a compromised LVEF of 40-45% [10]. Performing an open cholecystectomy was a life-saving measure; extreme attention was paid to minimize operative time and ensure continuous hemodynamic monitoring and support, as advised by the cardiac and anesthesia teams. 

Resolving the acute intra-abdominal infection was the top priority; this meant postponing the planned CABG until the patient’s condition stabilized post-cholecystectomy [11]. Acknowledging the high-risk nature of the surgery posed by the patient’s significant cardiac comorbidities, the multidisciplinary team implemented meticulous planning to optimize the perioperative management. The histopathological findings revealed acute necrotizing cholecystitis with extensive hemorrhage and neutrophilic infiltration, further confirming the condition’s severity (Figure 4), and consistent with findings reported in other cases of complicated ASC [12]. 

The favorable result, achieved through an open cholecystectomy followed by the subsequent percutaneous coronary intervention for the underlying severe 3VCAD, highlights the significant value of a well-coordinated, stepwise, and multidisciplinary approach in managing such complex and high-risk patients. The patient’s improved health and the resolution of the acute infectious process following cholecystectomy justified the decision to pursue PCI with stenting rather than proceeding with the initially planned CABG, allowing for a less invasive cardiac intervention with potentially lower immediate risks in this specific context [5]. 

In this case, we highlighted the importance of shared decision-making, especially in scenarios involving significant risks and complex management strategies [6]. The decision to proceed with open cholecystectomy was a crucial aspect of the management process, which was hard to be considered without the efficient collaboration between the general surgery, cardiology, cardiothoracic surgery, interventional radiology, anesthesiology, and ICU teams, which was essential to achieve a favorable outcome in a case with this complexity. 

## Conclusions

In conclusion, we have shown through this article the difficulties encountered while treating a patient suffering from severe cholecystitis and a perforated gallbladder that was presented in a complex status consisting of recent heart problems and preexisting cardiac conditions. The treatment process was divided into different approaches, starting with a conservative medical approach, progressing to a percutaneous drainage procedure, and ending with a critical open cholecystectomy. Through this multi-stage plan, we effectively tackled the severe abdominal infection while prioritizing the patient's cardiac well-being. After the surgery, the patient underwent a successful percutaneous coronary intervention, which stabilized his heart condition and eliminated the need for more invasive bypass surgery. Through our experience in this case, we would like to confirm the significance of personalized treatment plans that suit each patient's specific circumstances, promoting collaboration among medical specialties, and involving multi-specialty physicians in the decision-making when managing high-risk scenarios.
